# TbMgNi_4–*x*_Co_*x*_–(H,D)_2_ System. II: Correlation between Structural and Magnetic Properties

**DOI:** 10.1021/acsomega.3c04879

**Published:** 2023-08-09

**Authors:** Valérie Paul-Boncour, Premysl Beran, Charles Hervoches, Vitalii Shtender

**Affiliations:** † Université Paris-Est Créteil, CNRS, ICMPE, UMR 7182, F-94320 Thiais, France; ‡ Nuclear Physics Institute, Czech Academy of Sciences, 25068 Rez, Czech Republic; § European Spallation Source, ESS ERIC, Box 176, SE-221 00 Lund, Sweden; ∥ Department of Chemistry—Ångström Laboratory, Uppsala University, Box 538, 75121 Uppsala, Sweden; ⊥ Department of Materials Science and Engineering, Uppsala University, 75103 Uppsala, Sweden

## Abstract

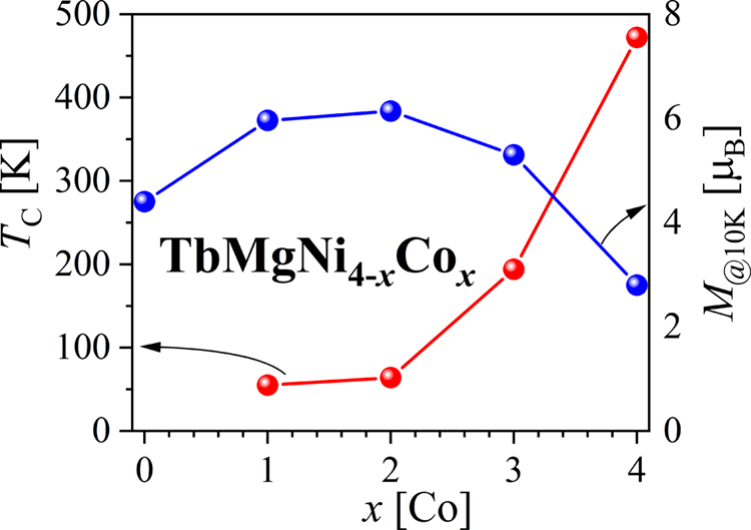

The magnetic properties of TbMgNi_4–*x*_Co_*x*_ intermetallic compounds and selected hydrides and deuterides of this system have been studied by various techniques, including magnetic measurements, in situ X-ray and neutron powder diffraction. The intermetallic compounds crystallize in a SnMgCu_4_-type structure and magnetically order below a Curie temperature (*T*_C_), which increases exponentially with the Co content. This can be due to the ordering of the Co sublattice. On the other hand, the insertion of D or H in TbMgNiCo_3_ strongly decreases *T*_C_. The X-ray diffraction measurements versus temperature reveal cell volume minima at *T*_C_ for the compounds with *x* = 1–3 without any hints of the structure change. The analysis of the neutron diffraction patterns for the intermetallics with *x* = 2 and 3 indicates a slightly canted ferrimagnetic structure below *T*_C_. The Tb moments refined at 16 K are 4.1(2) μ_B_/Tb for *x* = 2, and 6.2(1) μ_B_/Tb for *x* = 3, which are smaller than the free ion value (9.5 μ_B_/Tb). This reduction can be due to the influence of temperature but also reveals the crystal field effect. As Ni and Co occupy statistically the same Wyckoff site, an average Ni/Co moment was refined, leading to 1.7(2) μ_B_/atom for *x* = 2 and 1.8(1) μ_B_/atom for *x* = 3 at 16 K. This moment is slightly canted compared to the Tb moment.

## 1 Introduction

Many alloys and intermetallic compounds (IMCs) are considered for their properties in various energy-related applications such as hydrogen storage. This is dictated by the need for a sustainable future. Since IMCs are often termed functional materials owing to their superior properties in different fields, it makes such materials of great importance for investigation.^1^

In general, the formation of an extended homogeneity range for RMgNi_4–*x*_Co_*x*_ (R = rare earth) compounds is defined by (i) the existence of binary RT_2_ compounds (T = Ni or Co), which is a key point in the formation of RMgT_4_ = R_0.5_Mg_0.5_T_2_ IMCs, and (ii) the ability of the Ni and Co to replace each other in a homogeneity range, which depends on the nature of the R atom (the lighter R atom, the less Co substitution is possible). These compounds crystallize in a SnMgCu_4_-type structure, an ordered derivative of AuBe_5_. The last one is closely related to the MgCu_2_-type structure (C15) that is common for RT_2_ compounds.^2^

The hydrogenation properties of the R_1–y_Mg_*y*_Ni_4–*x*_Co_*x*_ compounds can be modified by changing the concentration of a specific element in the formula (Mg and Co) while keeping the same structure as the parent compound. The increase in hydrogen capacity and kinetics during solid–gas hydrogenation by Co substitution was first reported for YMgNi_4–*x*_Co_*x*_ (*x* = 0, 2, and 4) compounds.^3^ Similar results were obtained for RMgNi_4–*x*_Co_*x*_ (R = Nd^4^ or Tb,^5^ with *x* = 0–3 for Nd, and up to 4 for Tb), La_1–*y*_R_*y*_MgNi_4–*x*_Co_*x*_ (R = Pr^6^ or Nd,^7^*y* = 0 or 0.5, *x* = 0–3), La_1–*y*_R_*y*_MgNi_4–*x*_Co_*x*_ (R = Y or Ce, *y* = 0 or 0.5, *x* = 0–2),^8^ and (R, R′)_2–*y*_Mg_*y*_Ni_4–*x*_Co_*x*_ (R, R′ = Pr, Nd; *y* = 0.8–1.2; *x* = 0–2)^9^ compounds. As for the Mg content in (R, R′)_2–*y*_Mg_*y*_Ni_4–*x*_Co_*x*_ compounds, it should be larger than *y* = 0.8 to avoid amorphization. Also, lowering the Mg content decreases the equilibrium pressure.^9,10^ The studies of the electrochemical properties of such R_1–*y*_Mg_*y*_Ni_4–*x*_Co_*x*_ compounds did not demonstrate such an outstanding improvement of the hydrogen capacity versus the Co content. Only a small amount of Co (*x* = 0.5 for La_1–*y*_R_*y*_MgNi_4–*x*_Co_*x*_ (R = Pr or Nd, *y* = 0 or 0.5);^6,7^*x* = 1 for La_1–*y*_R_*y*_MgNi_4–*x*_Co_*x*_ (R = Y or Ce, *y* = 0 or 0.5);^8^*x* = 0.33 for LaMgNi_4–*x*_Co_*x*_^11^) slightly increases the discharge capacity while further increasing the Co content leads to the deterioration of the electrochemical properties. Additionally, Pr or Nd for La substitution improves the cyclic stability of the corresponding electrodes.^6,7^ On the other hand, electrode materials with a larger Mg content (*y* = 1.2) are characterized by improved electrochemical capacity due to both weight lowering and enhanced solid–gas H capacity.^9^

Hydrogen storage properties of TbMgNi_4–*x*_Co_*x*_ (*x* = 0–4) IMCs have been previously studied.^5^ It was shown that Co for Ni substitution allows: (i) a substantial increase of hydrogen capacity by at least 40% (TbMgNi_4_H_4_ vs TbMgCo_4_H_6_); (ii) a lowering of the equilibrium plateau of hydrogen pressure (from 1.1 down to 0.1 MPa H_2_, respectively); (iii) an improvement of the kinetic of hydride formation (reaction rate constant changes from ln(*k*) = −9.38 up to −6.12). Furthermore, it was found that the hydrogenation process of TbMgNi_4–*x*_Co_*x*_ goes through two equilibrium plateaus for the concentration range *x* = 2–4, and presumably at higher pressures for *x* = 0 and 1. For the Ni-containing compounds, the TbMgNi_4–*x*_Co_*x*_H_*z*_ hydrides crystallize with orthorhombic (3.7 ≤ *z* ≤ 4.1) and cubic (5.2 ≤ *z* ≤ 6) structures, while for TbMgCo_4_H_*z*_, the monoclinic and cubic structures were found for the same range of H content, respectively. The structural stability of the hydrides increases with Co content and upon deuterium for hydrogen substitution. A decreasing enthalpy of formation of the alloys upon Co for Ni substitution and the inverse effect on the corresponding hydrides has been observed by DFT calculations (for *x* = 0, 2, 4) as well.^5^

However, there are only a few studies of the magnetic properties of RMgNi_4–*x*_Co_*x*_ IMCs. The RMgNi_4_ (R = La, Ce, Gd, Dy, Ho, Tm, Yb) compounds display a paramagnetic Curie–Weiss behavior, which is driven by the rare earth moments.^12,13^ NdMgNi_4_ and its hydride^4^ together with CeMgNi_4_^13^ and CeMgNi_2_Co_2_^14^ display a Pauli paramagnet behavior. Gd_1.12_Mg_0.88_Ni_4_ orders antiferromagnetically below a Néel temperature *T*_N_= 4.6(5) K.^13^ NdMgNi_2_Co_2_ orders magnetically below 50 K, whereas its hydride shows a Pauli paramagnet behavior.^4^

The present study is a continuation of our previous work on the hydrogenation and the structural and electronic properties of the TbMgNi_4–*x*_Co_*x*_–(H/D)_2_ system.^5^ Herein, our purpose is to elucidate the magnetic changes that are induced by Co for Ni substitution and hydrogen/deuterium insertion. The experimental results of magnetometry, X-ray powder diffraction (XRPD), and neutron powder diffraction (NPD) of selected compounds will be presented and discussed. To better understand the magnetic properties of the TbMgNi_4–*x*_Co_*x*_ pseudobinary compounds, and the influence of hydrogen/deuterium insertion, the new experimental results will be analyzed and compared with the literature data for similar compounds.

## 2 Experimental Methods

Starting materials for the preparation of TbMgNi_4–*x*_Co_*x*_ (*x* = 0–4) intermetallic compounds (IMCs) were ingots of Tb, Ni, and Co (all with purity ≥99.9%), and Mg powder (325 mesh, 99.8%). All TbNi_4–*x*_Co_*x*_ (*x* = 0–4) alloy precursors were prepared by arc melting under a purified argon atmosphere, then ground, mixed with Mg powder, pressed into pellets, and annealed. More details on synthesis can be found in our previous work.^5^ Compared with previous work, new batches of TbMgNi_2_Co_2_ and TbMgNiCo_3_ samples have been prepared for neutron diffraction with a slightly modified procedure. In the first step, a TbNiCo alloy precursor was prepared by arc melting in a purified argon atmosphere. The as-cast TbNiCo buttons were ground in an agate mortar and mixed with mixed NiCo-powder or Co-powder (all with purity ≥99.9% and 325 mesh) and Mg powder in specific proportions. 3 wt % excess of Mg was added to compensate for the evaporation loss at high temperatures. The NiCo powder was used to ensure the absence of magnetic impurities as it was noticed that using Tb(NiCo)_4_ precursor induces more magnetic impurities compared to the TbNiCo + NiCo precursor. The powder mixtures were pressed into pellets and placed into tantalum containers, which were further loaded into a stainless-steel autoclave and sealed under an Ar atmosphere. Then, the samples were heated to 1273 K and then cooled to 773 K (heating and cooling performed within 24 h). The final temperature was kept for 100 h, after which samples were quenched in cold water. Hydrogenation of the selected alloys was performed using a Sievert-type apparatus as in the previous work.^5^

Phase-structural analysis of the samples was carried out by powder X-ray diffraction using Bruker D8 diffractometers (Cu Kα radiation). The collected XRPD patterns were analyzed by the Rietveld method^15^ using FullProf software.^16^ For the low-temperature XRPD (LTXRPD), the powders were mounted on single-crystal Si sample holders and X-ray diffraction patterns were collected using a Bruker D8 Advance with monochromatized (Cu Kα_1_) radiation between 20 and 300 K. Sequential refinement of LTXRPD data has been done with the Topas 6 software.^17^

Inductively coupled plasma optical emission spectrometry (ICP-OES) was used to estimate the chemical concentration of each element in the alloys. The mass of 16 ± 2 mg of each sample was dissolved in 1 mL of nitric acid (nitric acid 65%, VWR) over 5 h, diluted 50 times with Milli-Q water containing 5% HNO_3_, and filtered with 0.2 μm syringe filters (Whatman) before measurement. Avio 500 Scott/Cross-Flow Configuration instrument was used for the ICP measurements. A calibration curve was formed for the measurements using a Multielement Calibration Standard (CPAchem). Concentrations of 0 (blank), 0.1, 1, and 10 ppm of the elements Tb, Mg, Ni, and Co were used to create a 4-point linear regression. All measured values are within a relative standard deviation (RSD) of 5%.

Magnetization measurements were carried out using an MPMS-5S Quantum Design SQUID magnetometer and a PPMS09 from Quantum Design using the ACMS option. The samples were placed in a gelatin capsule and fixed with glass wool. The known diamagnetic contribution of the gelatin capsule (−2.8 × 10^–12^ emu/T) is considered negligible compared to the sample magnetization. Isofield magnetization curves were recorded between 2 and 300 K with applied fields of 0.03T. The measurements were generally performed with decreasing temperature. Isotherm magnetization curves were measured with decreasing field from 9 to −0.02 T for selected temperatures between 2 and 300 K.

The neutron powder diffraction (NPD) data for TbMgNi_2_Co_2_ and TbMgNiCo_3_ compounds were collected at different temperatures above and below the magnetic transitions. The experiment was performed on the MEREDIT instrument at the Nuclear Physics Institute (NPI, Czech Republic). A mosaic Cu monochromator (reflection 220) providing neutrons with a wavelength of 1.46 Å was used. FullProf suite software^16^ has been used to refine the nuclear and magnetic structure of the measured neutron diffraction patterns. The symmetry analysis to determine the possible magnetic structures was performed using ISODISTORT^18,19^ and Bilbao Crystallographic Server.^20^ Finally, the magnetic structure was visualized using VESTA.^21^

## 3 Results and Discussion

### 3.1 X-ray Diffraction at Room Temperature

All of the studied IMCs crystallize in the cubic SnMgCu_4_-type structure, and their cell parameters increase with Co content (Table 1). However, the two new samples prepared for the NPD experiment present larger cell parameters (0.33%) than the previously studied alloys with the same nominal compositions (TbMgNi_2_Co_2_ s4 and s5, TbMgNiCo_3_ s6 and s7). This can be explained as follows: (i) the final Mg content can vary as a small excess of Mg has been introduced to compensate for the evaporation loss; (ii) different synthesis protocols have been used to obtain single-phase compounds. To determine the final chemical composition, we have conducted ICP measurements for all samples (energy-dispersive X-ray spectroscopy was discarded due to the serious peak overlap of Mg and Tb, as shown before^22^). From the ICP results, we can clearly observe that the Mg content in the two published samples (TbMgNi_2_Co_2_ s4 and TbMgNiCo_3_ s6) is notably lower than for new samples prepared for NPD (s5 and s7), which is closer to the nominal composition (Table 1). The lack of Mg is mainly compensated by Ni/Co excess, which has a smaller atomic size. As a result, we have rather reduced cell parameters for the previously published samples compared to the NPD samples.

**Table 1:** Crystallographic Parameters from Rietveld Refinements of XRPD Data Collected at RT Together with Curie Temperature (*T*_C_) and Saturation Magnetization (*M*_S_) at 10 K for TbMgNi_4–*x*_Co_*x*_ Compounds and Deuterides

			unit cell parameters [Å]						
no.	compound	space group	*a*	*b*	*c*	*V* [Å^3^]	composition from ICP	*T*_C_ [K]	*M*_S_ [μ_B_/f.u.] @10 K
s1	TbMgNi_4_	*F*4̅3*m*	7.0205(1)			346.02(1)	Tb_1.03_Mg_1.00_Ni_3.97_		4.4(1)
s2	TbMgNi_3_Co	*F*4̅3*m*	7.0333(1)			347.92(1)	Tb_1.04_Mg_0.94_Ni_3.08_Co_0.94_	55	5.96(2)
s3	TbMgNi_3_CoD_4.3_	*Pmn*2_1_	5.0088(3)	5.3969(3)	7.3194(4)	197.86(2)			
s4	TbMgNi_2_Co_2_	*F*4̅3*m*	7.0452(1)			349.69(1)	Tb_1.06_Mg_0.81_Ni_2.04_Co_2.09_	64	6.97(2)^d^
s5^a^	TbMgNi_2_Co_2_	*F*4̅3*m*	7.0603(1)			351.94(1)	Tb_0.99_Mg_0.92_Ni_1.96_Co_2.13_	70	6.14(2)
s6	TbMgNiCo_3_	*F*4̅3*m*	7.0500(1)			350.45(1)	Tb_0.99_Mg_0.85_Ni_1.03_Co_3.13_	194(5)	4.14(1)
s7^a^	TbMgNiCo_3_	*F*4̅3*m*	7.0731(1)			353.86(1)	Tb_1.04_Mg_1.01_Ni_1.10_Co_2.85_	169(5)	5.30(1)
s8^b^	TbMgNiCo_3_H_4.2_	*Pmn*2_1_	5.006(2)	5.430(2)	7.3985(2)	201.14(1)		65	
		*F*4̅3*m*	7.5062(5)			422.92(5)		15	
s9^c^	TbMgNiCo_3_D_5.4_	*F*4̅3*m*	7.4785(3)			418.26(3)		75/15	3.60(4)
s10	TbMgCo_4_	*F*4̅3*m*	7.0763(1)			354.33(1)	Tb_1.01_Mg_1.08_Co_3.91_	472	2.80(1)

a New batch of samples for neutron diffraction.

b Prepared with s7.

c Prepared with s6.

d 2 K.

Results of the crystallographic and magnetic properties of the studied compounds are gathered in Table 1. The TbMgNi_3_CoD_4.3_ deuteride was synthesized in terms of magnetic properties; however, it undergoes rapid decomposition to the parent compound. The results for the TbMgCo_4_ compound were added for comparison. More results for this compound, including magnetization and neutron diffraction experiments at high temperatures, will be presented in a separate publication.

### 3.2 Magnetic Properties of TbMgNi_4–*x*_Co_*x*_ Compounds

**Figure 1:**
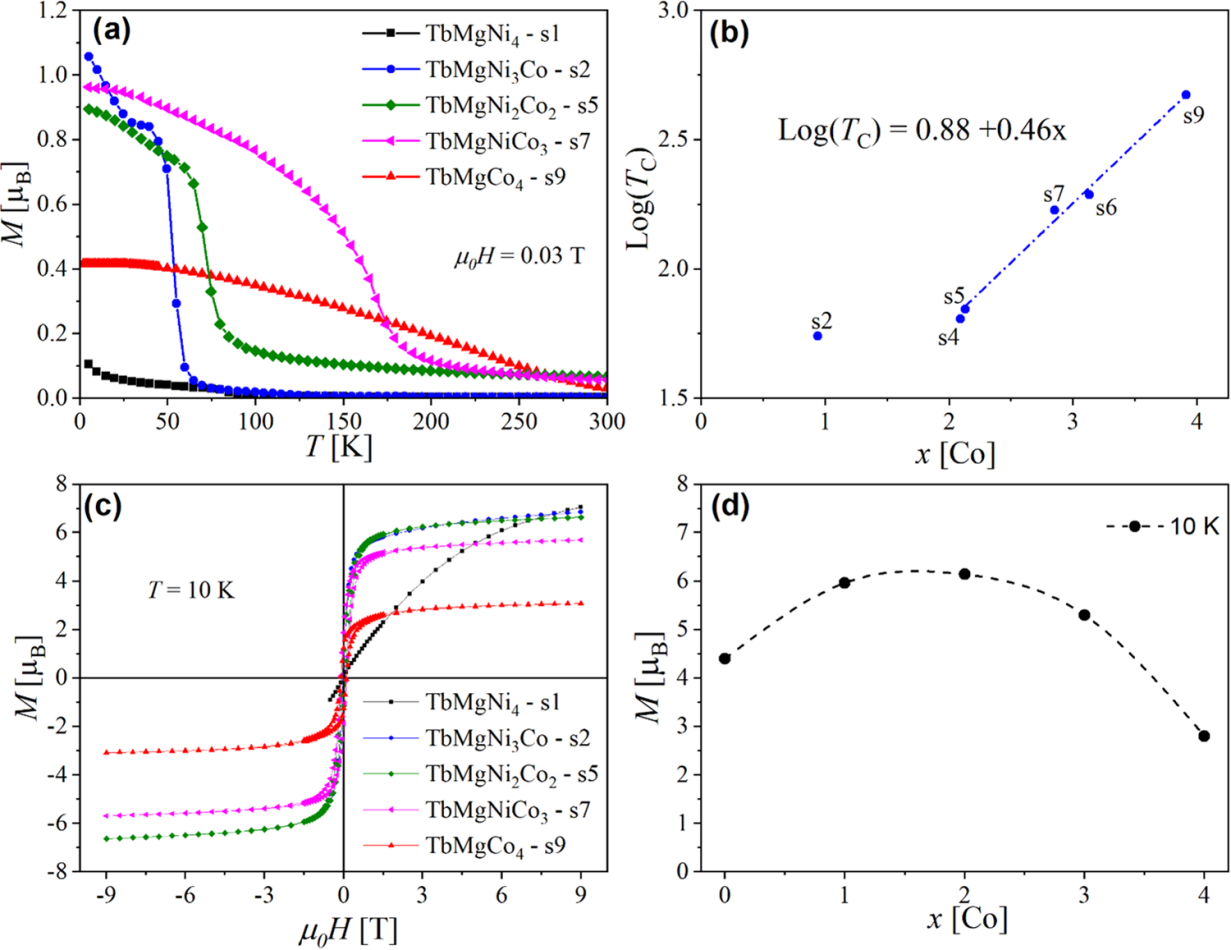
Magnetic data of TbMgNi_4–*x*_Co_*x*_ (*x* = 0–4) compounds. (a) *M*(*T*) at 0.03 T, (b) log(*T*_C_) versus Co content, (c) *M*(*H*) at 10 K, and (d) *M*_S_(*x*) at 10 K.

The magnetization curves of the TbMgNi_4–*x*_Co_*x*_ (*x* = 0–4) compounds as a function of temperature and an applied field of 0.03 T are compared in Figure 1a. The *M*(*T*) curve TbMgNi_4_ compound shows a paramagnetic behavior, while others demonstrate magnetic transitions which depend on Co concentration. The paramagnetic state of the TbMgNi_4_ compound agrees with a band structure calculation^5^ and with the results for other RMgNi_4_ compounds.^10,11^

The Curie temperature (*T*_C_), determined as the minimum of the *M*(*T*) first derivative, slowly grows versus Co concentration up to *x* = 2. A sharper increase is observed for *x* > 2, which can be fitted by a log(*T*) = *f*(*x*) linear variation (log(*T*) = 0.88 + 0.46*x*), indicating a strengthening of the magnetic interactions versus Co content (Figure 1b). Indeed, *T*_C_ is very sensitive to small Co variation: it is larger for sample TbMgNi_2_Co_2_ s5 versus s4 and for TbMgNiCo_3_ s6 versus s7 as they contain more Co. The magnetization of TbMgNi_4_ at 10 K increases progressively with the magnetic field and does not reach saturation at 9 T. On the contrary, for Co-containing compounds, the saturation is almost reached at 9 T, indicating a ferro- or ferrimagnetic behavior (Figure 1c). The saturation magnetization at 10 K also varies versus Co content in TbMgNi_4–*x*_Co_*x*_ compounds: at the beginning, it increases with a maximum at *x* = 2 and then decreases for a larger Co content (Figure 1d). This can be expected for a ferrimagnetic structure where Co moments are antiparallel to the Tb ones. Still, the magnetic structure needs to be fully solved by NPD to explain this variation. At 300 K, the magnetization of TbMgNi_4–*x*_Co_*x*_ (*x* = 0 and 1) shows linear behavior characteristic of a paramagnetic state. In contrast, the magnetization curves of the other compounds (*x* = 2–4), i.e., above their *T*_C_, contain a weak ferromagnetic contribution, possibly due to the segregation of magnetic impurities.

**Figure 2:**
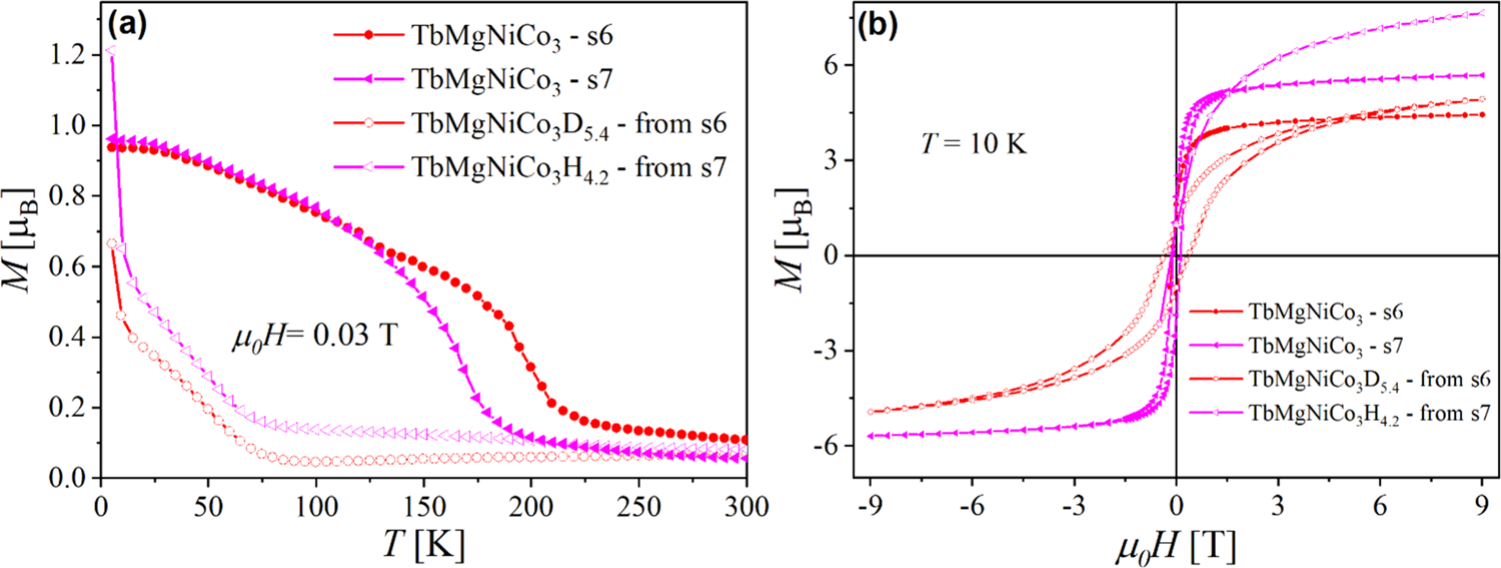
Comparison of (a) *M*(*T*) and (b) *M*(*H*) curves for TbMgNiCo_3_(H,D)_*x*_.

As the next step, the influence of hydrogen or deuterium insertion on the magnetic properties of the TbMgNiCo_3_ compound has been investigated. The magnetization curves *M*(*T*) of TbMgNiCo_3_H_4.2_ and TbMgNiCo_3_D_5.4_ are compared with those of their parent compounds in Figure 2a. Both hydride and deuteride display similar curves, with a reduction of *T*_C_ around 70 K compared to that of the pristine alloys. The hydride contains a mixture of orthorhombic and cubic hydrides, and for the deuteride, we expect a partial desorption of the cubic deuteride as the equilibrium pressure is around 7 bar. We can therefore attribute the transition at 65 K to the orthorhombic phase, whereas the increase of the magnetization below 30 K can be attributed to the cubic phase. For the deuteride, we also observed an increase in coercivity. It was observed that there is an increase in magnetization for hydrogenated samples compared to the parent compound, which can be related to the anisotropy induced by the orthorhombic distortion (Figure 2b). In any case, we can conclude that H/D absorption significantly reduces the magnetization, as it is often observed due to cell volume increase, which reduces the indirect Tb–Tb and Tb-T (T= d-transition metal) interactions. The modification of the density of states (DOS) by large H insertion was also shown to modify the DOS at *E*_F_ and, therefore, the magnetic moment of Co.

### 3.3 Neutron Diffraction for TbMgNi_2_Co_2_ and TbMgNiCo_3_ Compounds

**Figure 3:**
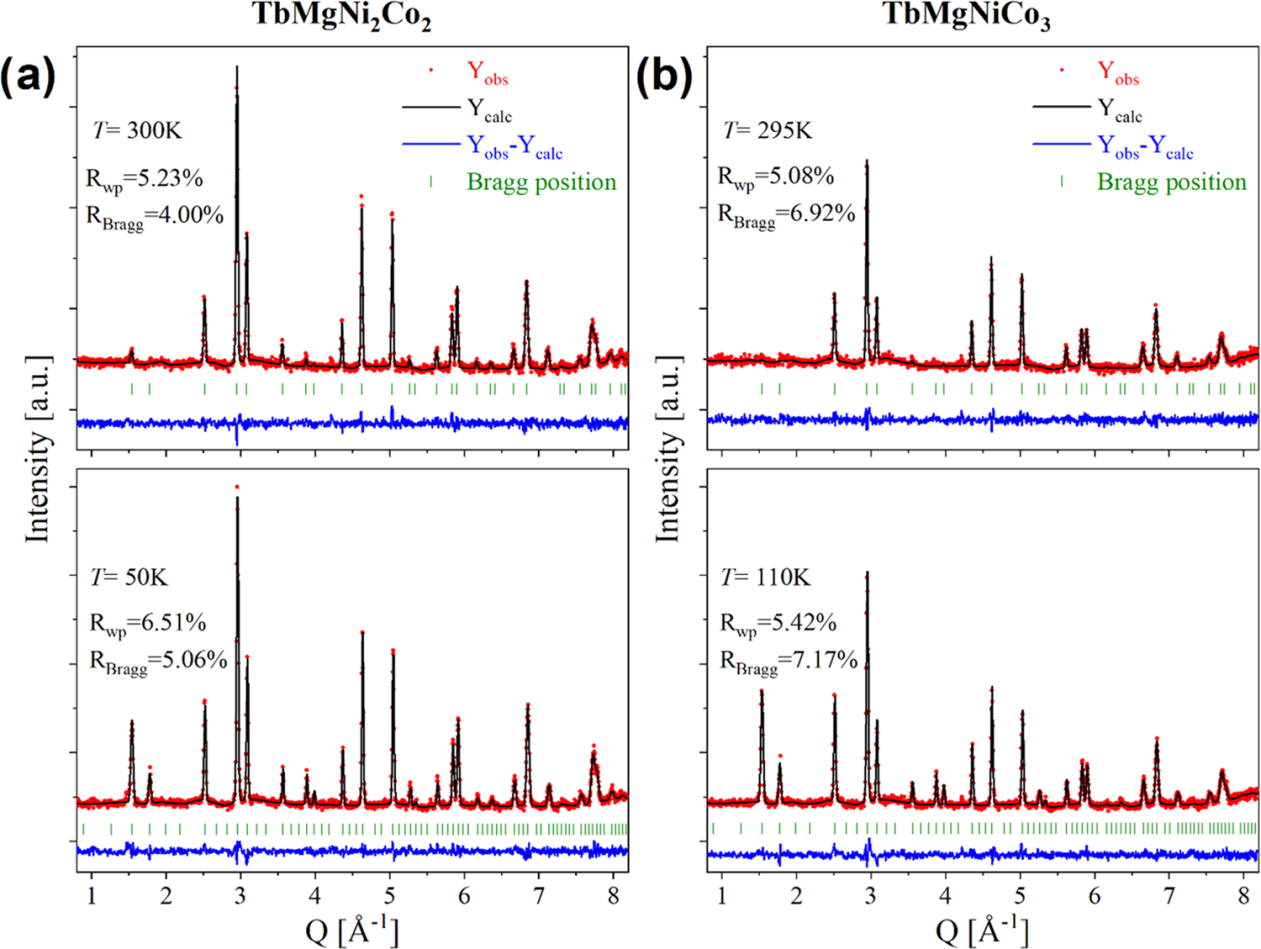
Rietveld refinement of the NPD patterns of TbMgNi_2_Co_2_ (s5) and TbMgNiCo_3_ (s7) compounds in the paramagnetic and ferrimagnetic state (a) at 300 and 50 K and (b) at 295 and 110 K, respectively.

To determine the magnetic structure of the IMCs, neutron powder diffraction (NPD) experiments were performed for TbMgNi_2_Co_2_ and TbMgNiCo_3_ compounds at different temperatures. As already mentioned, these new samples are single phase with unit cell parameters larger than those of the first batch of samples (Table 1). Their NPD patterns were measured in both paramagnetic and magnetically ordered states to solve the magnetic structure of the studied compounds. The evolution of the NPD patterns of TbMgNi_2_Co_2_ and TbMgNiCo_3_ at different temperatures between 16 and 300 K is presented in Figures 3a and 3b, respectively. At 300 K, the patterns can be indexed using the *F*4̅3*m* space group (Figure 3) with the ordered Tb and Mg atoms in 4*a* and 4*c* sites, respectively, and a random distribution of Ni and Co atoms in the 16*e* site forming the pyrochlore lattice. The presence of at least two types of magnetic atoms in the cubic structure triggers questions on how they coexist within the magnetic structure.

**Figure 4:**
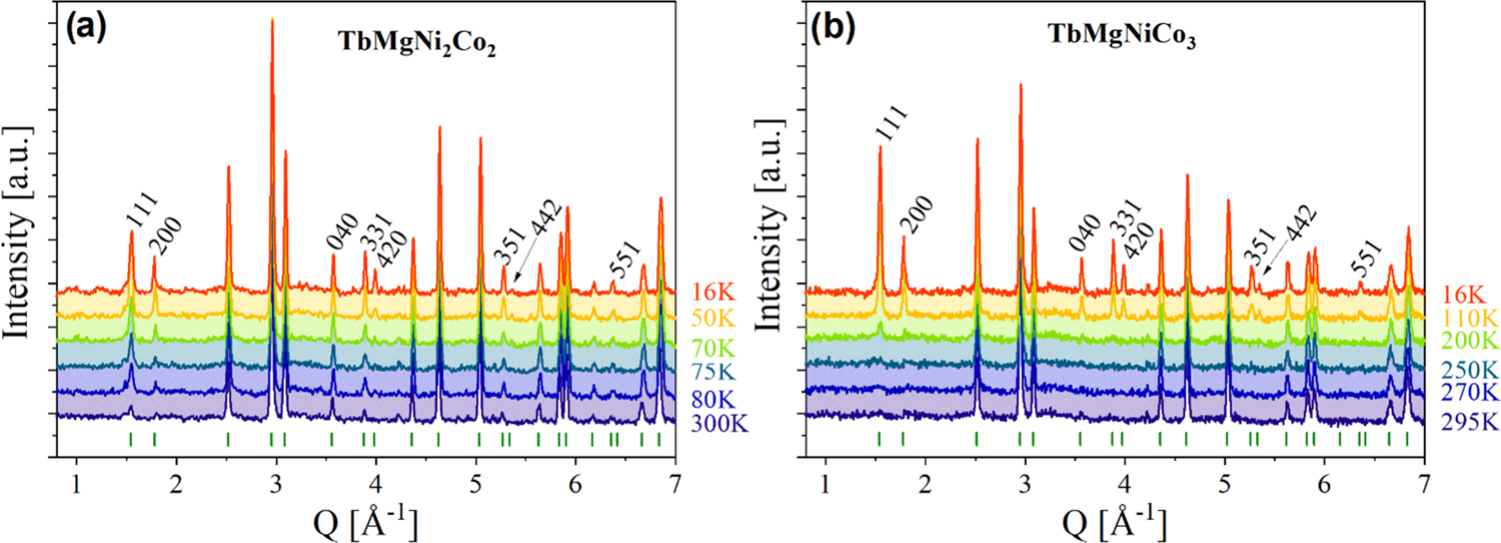
Evolution of the neutron diffraction patterns of (a) TbMgNi_2_Co_2_ (s5) and (b) TbMgNiCo_3_ (s7) compounds at different temperatures. Vertical bars denoted the Bragg peak positions. Indexed *hkl* peaks show the most pronounced magnetic contributions.

**Figure 5:**
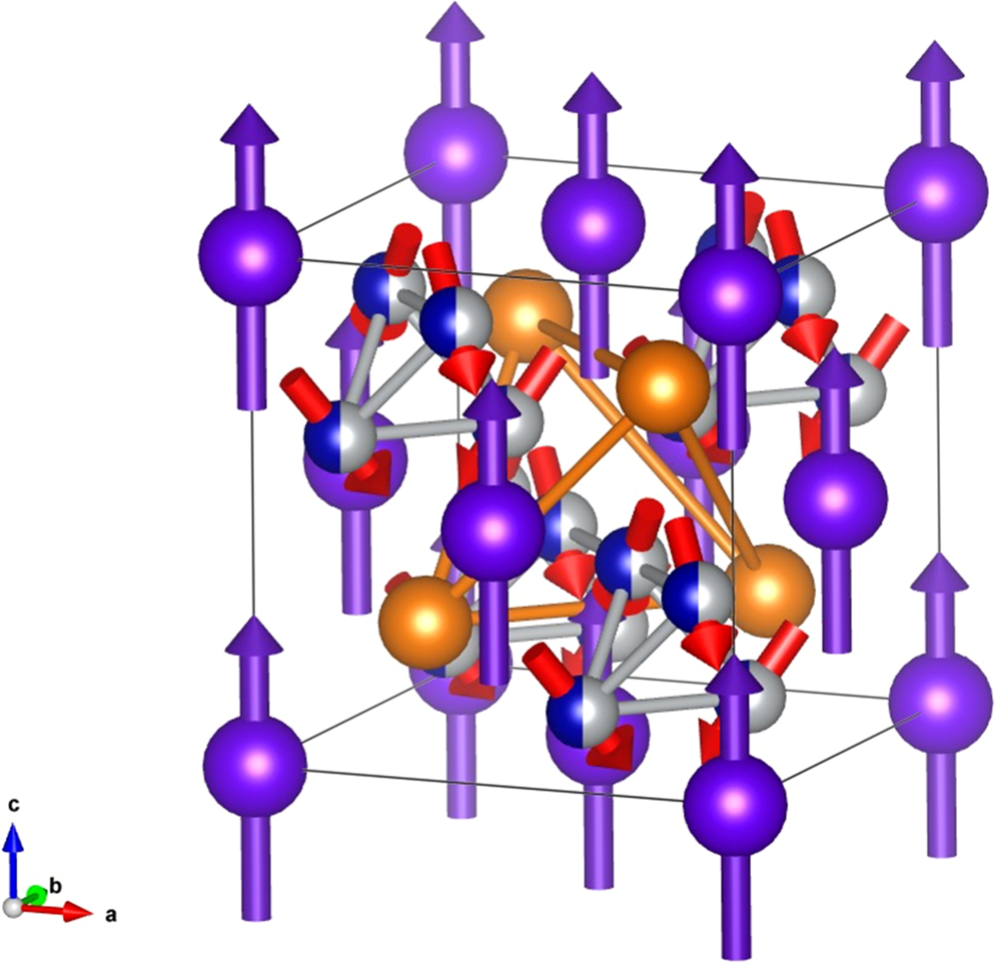
Magnetic structure of TbMgNi_2_Co_2_ at 50 K, with Tb atoms in violet, (Ni/Co) in blue/gray, and Mg in orange.

For both compounds, we observe below *T*_C_ an increase in the intensity of several Bragg peaks attributed to the contribution of the magnetic structure (Figure 4). These magnetic reflections can be indexed in the same cell as the nuclear structure, indicating that the magnetic propagation vector *k* is (0 0 0). The symmetry analysis suggested several possible magnetic space groups to describe the intensity changes of the magnetic Bragg peaks. The best fit was obtained with the magnetic space group *I*4^®^*m*′2′ (BNS 119.319) with the following relation to the parent structure: 1/2*a* – 1/2*b*, 1/2*a* + 1/2*b*, *c*. The refinement was performed within the parent unit cell setting, as displayed in Figure 3 for both compounds. The magnetic structure can be described as a quasi-colinear ferrimagnetic structure with Tb moments along the *c*-axis and antiparallel with the canted Ni/Co moments (Figure 5). As Ni and Co occupy the same crystallographic 16*e* site, the magnetic moments of both atoms were constrained to have the same values during refinement. The Tb moments in TbMgNi_2_Co_2_ (4.1(2) μ_B_/Tb at 16 K) are two times smaller compared to the Tb free ion value of 9.5 μ_B_. The average moment on the Co/Ni site is 1.7(2) μ_B_ at 16 K that is similar to 1.72 μ_B_ for Co-crystal.^23^ The Tb and Co/Ni moments in TbMgNiCo_3_ at 16 K are 6.2(1) and 1.8(1) μ_B_/atom, respectively. Assuming that the cobalt moment is near 2 μ_B_/Co and that of Ni, 0.6 μ_B_/Ni for both samples, we obtain M(Co/Ni) = 1.3 μ_B_ for *x* = 2 and 1.65 μ_B_ for *x* = 3 for a collinear arrangement. As a canting of the (Ni/Co) moment is observed, the values can vary. These results can also be summarized with the first principle calculations.^5^ The total calculated moments for TbMgNi_4_, TbMgNi_2_Co_2_, and TbMgCo_4_ were 0, 2.7, and 5 μ_B_/f.u., respectively. TbMgNi_4_ is paramagnetic as the nonpolarized configuration is more stable. The total moment increases for *x* = 2 and 4, and it shows the influence of Co substitution.

The evolution of the Tb and Co/Ni magnetic moments versus temperature for both compounds is depicted in Table 2, as well as the other refined parameters. For each compound, both magnetic sublattices order at the same temperature. The Tb and (Ni/Co) moments still observed at 200 K for TbMgNiCo_3_ can reflect a small variation of the transition temperature.

**Table 2:** Rietveld Refinement Results of the Crystal and Magnetic Structures of Cubic TbMgNi_2_Co_2_ (s5) and TbMgNiCo_3_ (s7) Compounds at Different Temperatures^a^

TbMgNi_2_Co_2_ (s5)						
*T* [K]	16	50	70	75	80	300
*a* [Å]	7.0417(2)	7.0411(1)	7.0406(1)	7.0403(1)	7.0407(1)	7.0582(2)
*V* [Å^3^]	349.17(2)	349.07(1)	349.00(1)	348.96(1)	349.02(1)	351.63(1)
*B*_iso_(Tb) [Å^2^]	0.74(2)	0.69(2)	0.85(2)	0.43(5)	0.45(2)	1.41(6)
*M*_Tb_ [μ_B_]	4.1(2)	4.4(1)	2.6(2)	2.2(2)	2.0(2)	-
*B*_iso_(Mg) [Å^2^]	0.1(−)	0.1(−)	0.11(1)	0.57(7)	0.57(7)	0.46(1)
Co/Ni *x*	0.6209(5)	0.6219(5)	0.6208(4)	0.6228(7)	0.6228(6)	0.6290(5)
*M*_Co/Ni_ [μ_B_]	1.7(2)	1.2(1)	0.8(1)	0.60(10)	0.65(11)	-
*B*_iso_(Co/Ni) [Å^2^]	0.1(−)	0.1(−)	0.11(1)	0.11(1)	0.11(1)	0.30(1)
*R*_p_ [%]	4.93	4.26	4.17	4.25	4.28	4.13
*R*_wp_ [%]	6.51	5.42	5.28	5.40	5.37	5.21
χ^2^	2.14	1.5	1.43	1.49	1.47	1.37

TbMgNiCo_3_ (s7)						
*T* [K]	16	110	200	250	270	295
*a* [Å]	7.0603(2)	7.0603(2)	7.0613(2)	7.0661(2)	7.0677(2)	7.0713(2)
*V* [Å^3^]	351.95(2)	351.95(2)	352.09(2)	352.82(2)	353.05(2)	353.58(2)
*B*_iso_(Tb) [Å^2^]	0.35(1)	0.49(1)	0.54(1)	0.54(4)	0.59(3)	1.07(9)
*M*_Tb_ [μ_B_]	6.2(1)	5.2(1)	1.3(3)			
*B*_iso_(Mg) [Å^2^]	0.35(1)	0.49(1)	0.54(1)	1.34(6)	1.39(6)	0.67(9)
Co/Ni *x*	0.626(2)	0.626(2)	0.625(2)	0.627(1)	0.625(1)	0.627(2)
*M*_Co/Ni_ [μ_B_]	1.8(1)	1.1(1)	0.7(1)			
*B*_iso_(Co/Ni) [Å^2^]	0.35(1)	0.49(1)	0.54(1)	0.46(1)	0.48(1)	0.51(2)
*R*_p_ [%]	4.91	4.27	4.29	4.15	4.05	4.04
*R*_wp_ [%]	6.34	5.42	5.41	5.26	5.07	5.14
χ^2^	1.99	1.47	1.48	1.39	1.30	1.33

a Both compounds have the space group *F*4̅3*m*, Tb at (0 0 0), Mg at (1/4 1/4 1/4), and Co/Ni at (*x x x*).

To explain the values of magnetic moments measured by NPD, a comparison can be made with previous work on R_3–*x*_Mg_*x*_Co_9_ compounds.^24^ It was found that the Tb_2_MgCo_9_ compound is ferrimagnetic with antiparallel and colinear alignment of Tb and Co moments. For the latter compound, the mean value for the Co moment is 1.67 μ_B_/Co, and Tb is assumed to be 9.5 μ_B_/Tb. Other compounds from the same series with light rare earth elements (R = Pr and Nd) have, as expected, a ferromagnetic behavior. The cobalt was estimated to have 1.67 μ_B_ in Tb_2_MgCo_9_, contrary to 0.9 μ_B_ in R_3–*x*_Mg_*x*_Co_9_ (R = Y, Pr, Nd) compounds, meaning that the molecular field induced by the Tb moment reinforces the Co moment. A similar trend was observed for the binary compounds in R–Co (R = rare earth metals) systems, where higher magnetic moments for the Co and lower for the heavy R were reported from magnetic measurements.^25^ Interestingly, for TbCo_2_ at 50 K, which displays a rhombohedral structure below *T*_C_ due to magnetostriction, the values obtained from NPD refinement were 8.30(5) μ_B_ for the Tb moment and 1.30(4), and 1.19(3) μ_B_ for Co1 and Co2 moments, respectively.^26^ The moments of the magnetic atoms in TbCo_2_ were assumed to be collinear and antiparallel. They are set to be along the *c*-axis of the rhombohedral structure, corresponding to the direction (1 1 1) in the cubic structure.^26^ For the TbCo_5_ compound, it was reported that the magnetic moments of Tb and Co atoms are antiparallel.^27^ These moments are aligned along the *a*-axis below 365 K, and along the *c*-axis above 450 K. Between these two temperatures, the magnetization axis rotates continuously. The cobalt atoms of the two crystallographic sites of this compound have slightly different magnetic moments, like the TbCo_2_ compound, but the actual value is around 1.7 μ_B_.

Notably, below *T*_C_, a small increase of unit cell parameter was observed for the TbMgNi_2_Co_2_ compound, while for TbMgNiCo_3_, it was nearly constant according to the calculation of the neutron data sets (see Table 2). To eliminate the effect of any structural changes in TbMgNi_2_Co_2_ and TbMgNiCo_3_ compounds, which could influence the observed values of the magnetic moments, low-temperature X-ray diffraction experiments were conducted.

### 3.4 Low-Temperature X-ray Diffraction for TbMgNi_4–*x*_Co_*x*_ Compounds

**Figure 6:**
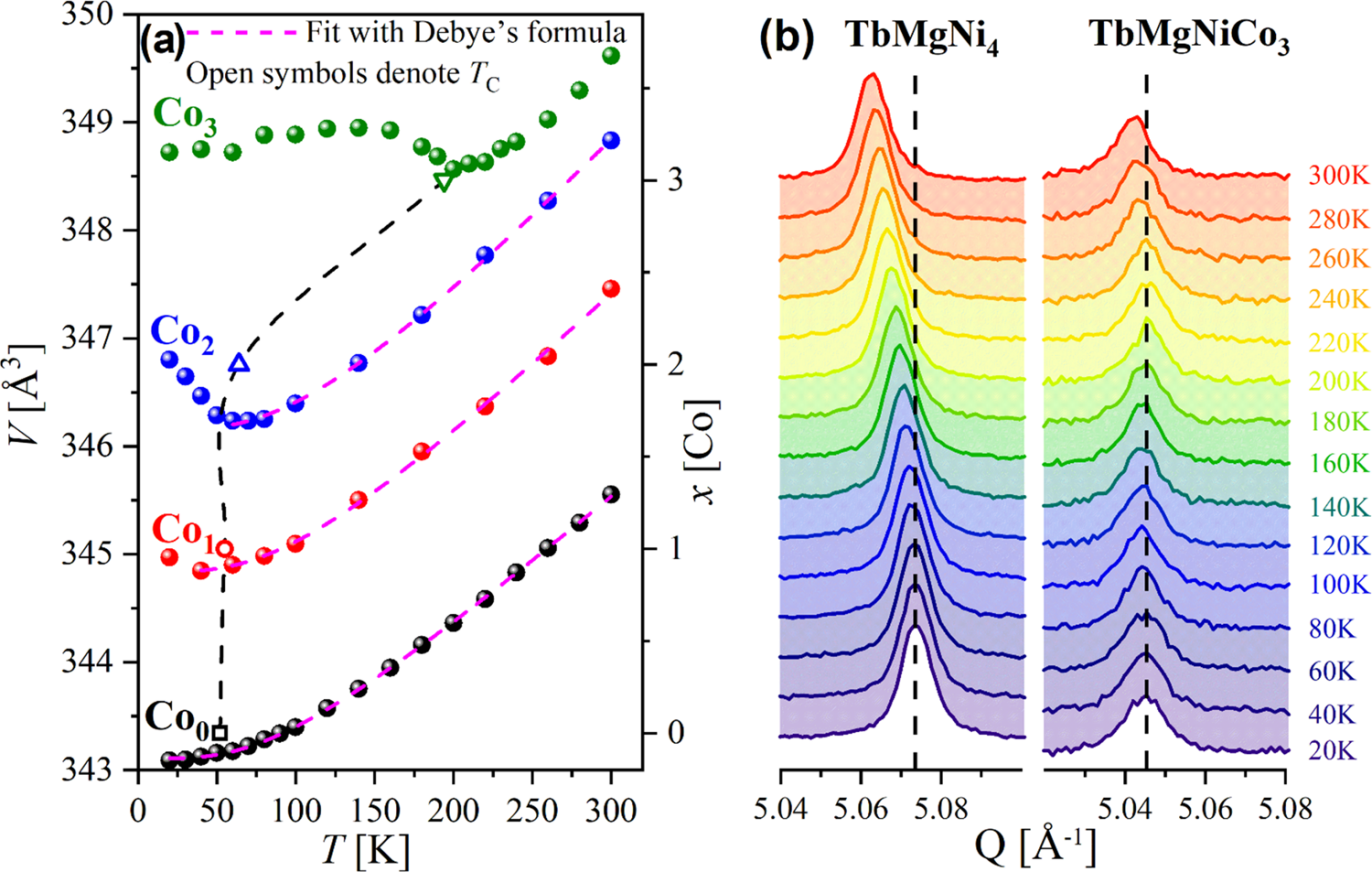
(a) Thermal evolution of unit cell volumes for TbMgNi_4–*x*_Co_*x*_ (*x* = 0–3) compounds (s1, s2, s4, s6). The general error bars of the calculated unit cell volumes are 0.02 Å^3^. Values of *T*_C_ coincide with the beginning of the increase of the unit cell volumes of the Co-based IMCs. (b) Comparison of the evolution of the 440 peaks for TbMgNi_4_ (s1) and TbMgNiCo_3_ (s6) compounds versus temperature (dashed lines are for the visual comparison).

For the low-temperature X-ray powder diffraction (LTXRPD), the samples from the previous study^5^ were used. As a result, very intriguing behaviors of the lattices were observed, which are consistent with NPD results. Namely, for all Co-containing specimens, an anomalous increase in the unit cell was noticed at low temperatures. The strong upturn of the unit cell volumes starts to appear exactly at the temperature of magnetic transition (*T*_C_). However, no crystal symmetry changes were detected, neither from XRPD (no splitting of the peaks through the entire temperature range) nor from the DSC experiment (any peaks were detected for TbMgNiCo_3_ in the range of 125–300 K). Dependences of the unit cell volumes as a function of temperature are presented in Figure 6a and can be analyzed using the Debye formula:^28^
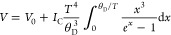
1where *V*_0_ is the unit cell volume at 0 K, *I*_C_ is the coefficient including the Grüneisen and compressibility parameters (for higher temperatures, the coefficient is the slope of *V*(*T*) dependence), and θ_D_ is the Debye temperature. Notably, all parameters expand with the increase of Co content (see Table 3).

**Table 3:** Structural Parameters of TbMgNi_4–*x*_Co_*x*_ (*x* = 0–3) Compounds at LTXRPD

no.	compound	*V*_20_ [Å^3^]	*V*_T_ [Å^3^]	*V*_0_ [Å^3^]	*I*_C_	θ_D_ [K]	*R*^2^
s1	TbMgNi_4_	343.09(1)		343.11(1)	0.0384(4)	347(7)	0.9998
s2	TbMgNi_3_Co	344.97(3)	344.85(3)	344.84(3)	0.0428(19)	382(32)	0.9988
s4	TbMgNi_2_Co_2_	346.80(1)	346.23(1)	346.15(2)	0.0474(16)	424(24)	0.9995
s6	TbMgNiCo_3_	348.42(2)	348.56(2)				

The *V*_T_ (unit cell volume at 0 and 20 K and at the temperature at which an increase of cell parameter is observed) (basically, at this temperature, we have the lowest value of *V*). *T*(TbMgNi_3_Co) = 40 K, *T*(TbMgNi_2_Co_2_) = 70 K, *T*(TbMgNiCo_3_) = 200 K. *I*_C_, and θ_D_ are refined parameters of eq 1.

LTXRPD data for the Co-free sample were fully described in eq 1, while for the others, the Debye formula was applied just above the deviation of cell parameters. For the TbMgNiCo_3_ compound, it was not possible to fit the data with this equation. The first thing which was noted is a coincidence of the cell volume jump at low temperatures with *T*_C_ for Co-containing samples, which can be due to magnetoelastic coupling (see Figure 6a). A comparison can be made with TbNi_2_ and TbCo_2_ compounds, as TbMg*T*_4_ compounds form a solid solution based on these compounds, for instance, TbCo_2_–TbMgCo_4_.^22^ For both binaries (TbNi_2_ and TbCo_2_), rhombohedral distortion was observed below *T*_C_.^29^ Changes of the unit cell volume with temperature were also observed for TbCo_2_,^26^ similar to what we observed for TbMgNiCo_3_. However, we could not identify any structural changes in the studied samples (see Figure 6b; LTXRPD data for the TbMgCo_4_ compound will be presented in a future paper). This can be somehow explained by the low resolution of the used X-ray diffraction technique, like in one of the studying related to Terfenol-D.^30^ Interestingly, the magnitude of the lattice distortion is proportional to the magnetoelastic coupling coefficients, which are usually very small. Thus, the structure change is often too small to be detected by the conventional XRD technique.^30^ However, the LTXRPD study for the Tb_3_Ga_5_O_12_^31^ compound also revealed an increase of unit cell parameters at lower temperatures. Such a thermal anomaly was correlated to the magnetic behavior and explained by the occurrence of a terbium orbital magnetic order for Tb^3+^.^31^ Additionally, we performed LTXRPD for the YMgNi_2_Co_2_^3^ compound and did not see any increase in the cell parameters at lower temperatures [unpublished data]. This confirms that the discontinuous changes of unit cell parameters are related to the nature of rare earth, as found for Tb_3_Ga_5_O_12_ vs Y_3_Ga_5_O_12_ or Tb_3_Ga_4_FeO_12_ vs Y_3_Fe_5_O_12_.^31^ In the end, for nonmagnetically ordered compounds (Tb_3_Ga_4_FeO_12_; Tb_3_Ga_5_O_12_; Tb_3_Al_5_O_12_), there was no structural distortion at low temperature (only abnormal thermal expansion of the unit cell), while cubic to rhombohedral distortion was evidence for magnetically ordered compounds (Tb_3_Fe_5–*x*_Ga_*x*_O_12_ (*x* = 0–2)). Considering all of the above statements, it can be concluded that the deviation from thermal expansion in TbMgNi_4–*x*_Co_*x*_ samples is induced by the Tb magnetic order. Tb is responsible for the increase of the unit cell at lower temperatures, and since TbMgNi_4_ is paramagnetic, we do not have such an increase of the unit cell.

## 4 Conclusions

In this work, the magnetic properties of TbMgNi_4–*x*_Co_*x*_ compounds have been investigated by combining magnetic measurements with X-ray and neutron powder diffraction measurements at different temperatures. The magnetic ordering temperature *T*_C_ increases versus Co content following a Logarithm law for *x* ≥ 2, which indicates that the magnetic ordering of Co is very sensitive to the number of Co neighbors. The NPD performed for *x* = 2 and 3 shows that they order below *T*_C_ in a slightly canted ferrimagnetic structure with the same ordering temperature for both Tb and transition metal sublattices. The Tb and mean (Ni/Co) moments also increase at 16 K versus the Co content. A deviation from linearity is observed in the cell volume variation at *T*_C_ for compounds that contain Co and is attributed to a weak magnetostrictive effect on Tb. H or D insertion significantly decreases the magnetic ordering temperature, as observed for TbMgNiCo_3_(H,D)_*x*_ compounds.

Finally, summarizing all results within the TbMgNi_4–*x*_Co_*x*_–(H,D)_2_ system, we can say that Co is a lever for the physicochemical properties that acts in a positive way. Co does the following: (i) a substantial increase of hydrogen capacity; (ii) a lowering of the equilibrium plateau of hydrogen pressure; (iii) an improvement of the kinetic of hydride formation; (iv) an increase of *T*_C_. As for magnetic behaviors, it varies and most probably depends on the statistically distributed Ni/Co atoms within the pyrochlore lattice. Magnetization of the compounds decreases with increasing Co content, which implies stronger Co/Tb competition even though the molecular field induced by the Tb moment reinforces the Co moment. All in all, it triggers the investigation of the magnetic structure of the TbMgCo_4_ compound where Co will solely occupy the pyrochlore lattice.
